# EcoRad: sustainable radiology and the ecology of economics

**DOI:** 10.1093/bjro/tzaf027

**Published:** 2025-10-26

**Authors:** Benjamin E Northrup, Kate Hanneman, Reed A Omary

**Affiliations:** Section of Musculoskeletal Imaging and Intervention, Mallinckrodt Institute of Radiology, Washington University School of Medicine, St. Louis, MO 63110, United States; Division of Cardiothoracic Imaging, Department of Medical Imaging, University Medical Imaging Toronto, University of Toronto, Toronto, ON M5G 2N2, Canada; Department of Radiology, Vanderbilt University Medical Center, Nashville, TN 37232, United States

**Keywords:** sustainability, medical economics, planetary health, supply chain, carbon pricing

## Abstract

This review explores the dual meaning of the prefix “eco”—ecology and economics—and the transformative idea of synthesizing the two into a single “eco” framework. This framework gives rise to EcoRad, which blends economic and ecologic principles to optimize radiology practice. EcoRad strives to achieve the triple bottom line by approaching economic challenges from a planetary health perspective and by using economic approaches to enhance planetary health. In effect, this expands the traditional focus on financial performance to also include social and environmental impact. With EcoRad as a guide, radiology departments are called upon to consider 5 actions that can help overcome barriers to sustainable radiology: adopt sustainable procurement and maintenance, integrate green information technology (IT) and operational efficiencies, advocate for payment models that reward green radiology, champion green budgeting, and involve patients, industry, third-party payors, and policymakers in sustainability.

## Introduction

As sustainability in radiology advances, economic barriers to implementing environmentally sustainable practices must be overcome. These barriers, including *status quo* bias, sunk cost fallacy, and prioritizing short-term profit and growth, lead to perceptions of higher cost for sustainable practices and decreased value of long-term benefits.^1–3^

Understanding the interplay of ecology and economics is key to overcoming these obstacles. Furthermore, although these barriers are oft cited, the goals of economic responsibility and green radiology greatly overlap.

In this review, we explore the intersection of ecology and economics in radiology, which we term the “eco” framework. We introduce EcoRad, underpinned by the “eco” framework, and its triple aim of planetary health, financial stewardship, and improved patient care.^4^^,^^5^ Subsequently, we discuss the complementary approaches of EcoRad: solving economic challenges with the tools of planetary health and using principles of economics to enhance planetary health. Our objective is to highlight actions that can overcome barriers to sustainable radiology.

## Defining ecology and economics

The root term “eco-” has grown to embody 2 powerful ideas, at once separate and interconnected: ecology and economics. Derived from the Greek word *oikos*, “eco” originally meant “house” or “environment.” Ecology thus becomes the study of interactions of organisms with the environment, and economics, literally, management of the house. However, the term economics has broadened in modern times to refer to a social science that studies the production, distribution, and consumption of goods and services. This encompasses how societies and individuals allocate scarce resources.

Much like the root “eco-,” the term “sustainability” also bridges financial and environmental concepts. Although the economic definition, referring to the ability of an entity to support a defined level of economic production indefinitely, can stand alone, this depends upon environmental sustainability, the responsible stewardship of natural resources, and the environment to ensure that the needs of current and future generations can be met.

**Figure 1.**
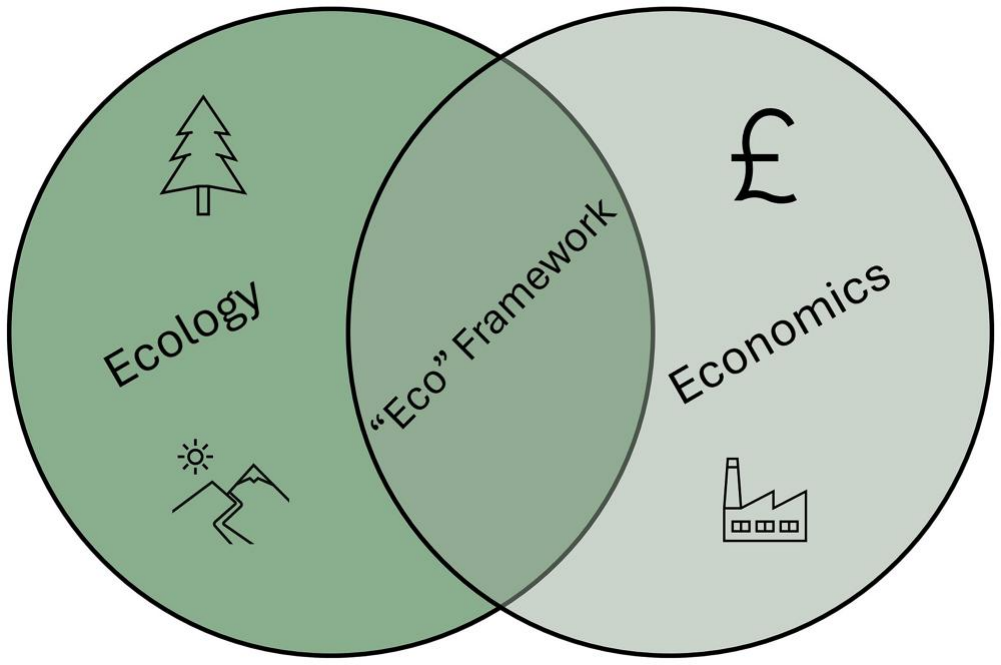
The “eco” framework.

The definitions of ecology and economics intersect in several ways, forming the “eco” framework (Figure 1). First, they both emphasize the interdependence of individuals with their system or environment. Second, the production, distribution, and consumption of goods and services depend upon resource availability, weather patterns, and the absence of natural disasters or environmental degradation. Ecologic principles can thus be embedded into economic decision-making. In so doing, the value of preserving ecosystems can be included along with environmental costs.^6^

**Figure 2.**
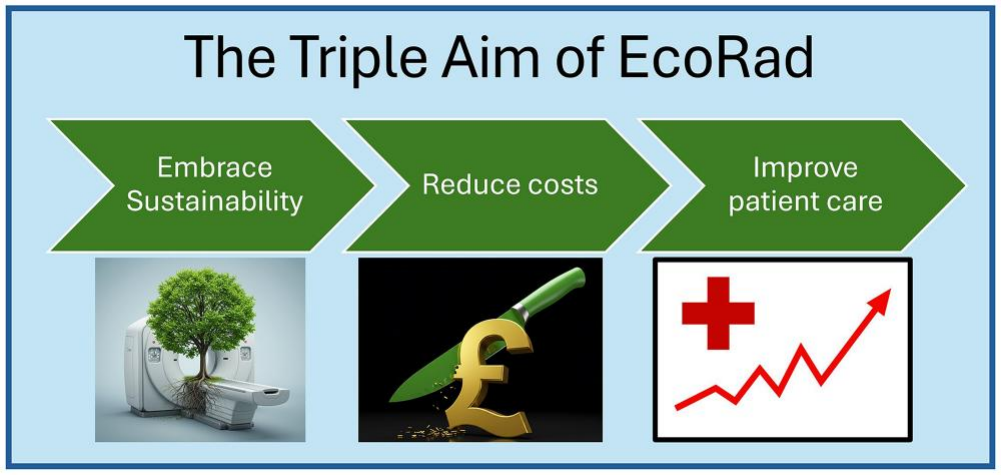
The triple aim of EcoRad.

While there are potential trade-offs between economic and ecological goals, the traditional boundaries between these 2 realms can be diminished by applying the toolkit of ecology to solve economic problems and vice versa. When this approach is applied to radiology practice, we propose the moniker EcoRad. The goal of EcoRad is to embrace the triple aim of enhanced sustainability, reduced costs, and improved patient care (Figure 2).

## A planetary lens on the economics of radiology

Rapid technological advances in radiology have improved diagnostic exams and image-guided procedures, yet these have come with soaring costs^7^ and escalating environmental impact, due in large part to increasing volumes. Delivery of radiology services consumes vast amounts of energy, requires finite resources such as iodine, gadolinium, and helium, and generates significant greenhouse gas (GHG) emissions and waste.^8–10^ Specifically, as technology has improved and demand for cross-sectional imaging and interventional radiology services has increased, energy use and waste has surged.

On the other hand, the environment also impacts radiology services. Environmental exposures, such as air pollution, extreme heat, and toxic chemical contaminants, have been increasingly linked to multiple health conditions, including cardiovascular and respiratory diseases.^8^^,^^11^ These diseases often necessitate diagnostic imaging for evaluation, leading to heightened utilization of radiology services.^12^ This uptick in imaging not only escalates healthcare costs but also amplifies the environmental impact of radiology departments.^13^ Beyond increased imaging demand, environmental exposures can also directly impact critical infrastructure in radiology, leading to substantial economic costs. Extreme weather events, such as hurricanes and floods, can damage imaging equipment, disrupt power supplies, and necessitate costly repairs or replacements. For instance, Hurricane Maria severely damaged Puerto Rico’s healthcare infrastructure, including radiology services, leading to prolonged service disruptions and increased operational costs.^14^^,^^15^ The primary drivers of disruption of radiology services are damage to infrastructure and supply chain interruptions. This results in excess mortality, increases the cost of care, and exacerbates healthcare inequity.^16–18^

These challenges provide opportunities for the EcoRad approach. By embracing both economic and ecologic responsibility (Table 1), EcoRad can advance the triple aim.

**Table 1.** The ecologic and economic components of the EcoRad approach.

Components	Ecologic	Economic
Energy efficiency	Reduced greenhouse gas emissions	Reduced electricity costs
Resource management and waste reduction	Conservation of raw materials, reduced landfill burden, decreased Scope 3 emissions	Decreased costs for supplies and equipment, storage of waste, and disposal of waste
Water conservation	Reduce strain on local water resources, lower energy use for water treatment and transportation	Reduced water and sewer overhead
Sustainable procurement	Encourage industry to adopt green practices, reduced environmental impact throughout products’ lifecycles	Reduction in total cost of ownership (includes energy use, maintenance, disposal)
Workflow optimization	Decreased unnecessary energy and resource use, reduce emissions from patient and staff travel	Increased throughput, decreased cost of errors and duplicated work, reduced burnout
Waste segregation and recycling	Diversion from landfills, facilitation of reuse and recycling, correct handling of hazardous materials	Reduced disposal costs, downstream benefits of reuse and recycling
Adaptation, resilience, and preparedness	Prepare for and minimize the effects of climate change and build capacity to recover quickly from climate-related environmental impacts	Reduce financial losses from infrastructure damage and rising demand for imaging

Viewing radiology through a planetary lens requires recognizing that every imaging procedure, every piece of equipment, and every operational decision contributes an ecological footprint. This impact can be quantified comprehensively using an environmental life cycle assessment (LCA), as has been undertaken at a large US medical centre.^19^ Additional previous studies have used LCAs to analyse energy use of imaging equipment, monitors, and other portions of the value chain.^20–27^ When coupled with cost-effectiveness analyses,^28^ LCAs can help radiologists prioritize EcoRad components to target. Other methods to estimate ecologic impact include waste audits, power metre measurements, modelling studies, and environmental impact assessments.^29–31^ These studies show that imaging equipment and operations are the most impactful categories, economically and environmentally. Nearly all radiology departments can target these categories in the following ways:

### Imaging equipment

Through energy efficiency and sustainable procurement, transportation, resource management, and waste reduction, EcoRad can reduce the high costs and environmental impact of imaging equipment.

An entity’s emissions are divided into 3 scopes (Table 2) by the Greenhouse Gas Protocol.^32^ Unlike the rest of healthcare, a substantial proportion of radiology emissions are Scope 2. MRI services account for approximately half of overall diagnostic radiology emissions, 79% of which are Scope 2. Scope 2 emissions account for 42% of the total for CT and 47% for radiography/fluoroscopy.^19^ Thus, reducing energy use offers the greatest dual benefit for imaging equipment, mitigating environmental impact and costs. Accordingly, energy efficiency is the first component of EcoRad. In one recent example, Woolen et al^33^ show that MRI protocol acceleration reduces energy use, emissions, and costs, while also increasing imaging capacity and revenue. Other options to improve energy efficiency in radiology include powering down imaging equipment when not in use, abbreviating imaging protocols, and considering lower energy modalities over higher energy modalities when clinically appropriate.^34^^,^^35^

**Table 2.** The 3 scopes of the Greenhouse Gas Protocol.

Scope	Definition	Examples
Scope 1	Direct emissions, from owned or controlled sources	On site emissions, anesthetic gases, fleet travel
Scope 2	Indirect emissions, from producer of energy purchased	Purchased electricity
Scope 3	Indirect emissions, upstream and downstream, excluding Scope 2	Supply chain, emissions related to water use and waste disposal

Supply chain emissions are also significant, with Scope 3 emissions accounting for the majority of emissions across healthcare. An analysis of the carbon footprint of the National Health Service (United Kingdom) in 2019 showed that 62% of healthcare emissions were from supply chain.^36^ The UK Green Building Council and the United States Environmental Protection Agency^37^^,^^38^ provide resources to reduce supply chain emissions.

Traditionally, imaging equipment has been produced with limited consideration for energy efficiency, water use, durability, or end-of-life repurposing. By working with vendors to shift to a circular economy, radiology departments can reduce their environmental impact. The circular economy aims to extend the life of imaging equipment and reduce waste to a minimum through sustainable design, reuse, repair, refurbishment, and recycling.^15^

Industry partners have already built the foundation for these future collaborations. For instance, many vendors have issued environmental product declarations. These detail the environmental impact of a product in a single report, which is useful for purchasing decisions.^22^ Additionally, routine maintenance, such as software updates and hardware upgrades, can improve operational efficiency and extend the life of existing equipment. Doing so reduces financial and carbon costs, while optimizing planning for replacement.^39^^,^^40^

### Clinical operations

Operational efficiencies and workflow optimization are central tenets of EcoRad. The greatest triple benefit from an operations standpoint is to reduce inappropriate medical imaging. Inappropriate imaging produces an average of 3.55-129.2 kT CO2e per year (among United States Medicare Part B beneficiaries only, on average, about 60 million people) and costs over $12 billion per year.^41^ The emissions estimate’s wide range is largely due to heterogeneity in the emissions profiles of CT and MR scanners. Furthermore, inappropriate imaging can lead to downstream morbidity, due to sequelae of radiation exposure or complications of unnecessary procedures.

Operational efficiencies related to medical images have long significantly influenced the budgets and environmental footprints of radiology departments. Although the advent of digital imaging has reduced the waste associated with chemical processing and physical storage of films, digital systems require robust data centres that consume significant energy and water.^42^ Worldwide, data storage accounts for 2% of electricity consumption, similar to CO_2_ emissions of the airline industry.^43^ Promoting green data centre and information technology (IT) polices—including energy-efficient servers; water-efficient cooling systems; cloud computing solutions; optimized data management with compression techniques and regular data purges; and electronic waste recycling—can reduce this environmental impact, while also lowering costs.^44^

To further increase efficiency, “just-in-time” scheduling can streamline operations and reduce idle time for expensive equipment. However, such improvements can be hindered by “did not attend” or patient no-show behaviour. Artificial intelligence (AI)-based appointment systems offer a potential solution, using past patient data and demographic characteristics to identify patients with a high likelihood of missing appointments, and offering backup appointments or alternative time slots.^45^ These algorithms minimize energy waste and maximize resource utilization. By integrating these changes as part of the EcoRad approach, radiology departments benefit their bottom line, improve efficiency, and promote patient-centred care.

## Integrating economic frameworks to enhance planetary health

Conversely, applying economic frameworks to planetary health can balance the cost of radiologic care with environmental stewardship. Economics has traditionally overlooked the hidden costs of environmental degradation. Instead, we recommend accounting for the external costs of ecological damage and pollution.

### Green budgeting and carbon pricing

For instance, “green budgeting” can be incorporated into financial planning. This involves tracking both the direct financial and the indirect environmental costs associated with energy use, waste generation, and resource depletion. For example, carbon pricing can monetize emissions and include them in cost-effectiveness analyses.^46^^,^^47^ Carbon pricing has its underpinnings in the framework of ecological economics, which theorizes that implementation of policies such as carbon pricing requires separation of the 3 basic economic goals of efficient allocation, equitable distribution, and sustainable scale.^48^ By assigning a value to these currently hidden costs, radiology leaders can more accurately compare the long-term benefits of investing in sustainability to the *status quo*.

### Resource scarcity and resilience

Energy is a scarce resource which can be affected by extreme weather.^49^ Disruptions halt the generation of energy and add significant costs for repair, replacement, and insurance. Adding new sources of clean energy can bolster energy security. For instance, distributing energy through clean microgrids can limit the untoward effects of disrupted central grids. Other ways to increase resilience to extreme weather events include improved battery technology, integrating environmental threats into existing cybersecurity, pandemic, and supply chain management plans, and upgrading radiology infrastructure to minimize damage from power outages and other environmental events.^50^^,^^51^

Medical imaging is also highly dependent on a number of scarce resources, including helium, iodine, and gadolinium. Economic principles, including evolutionary economics and the circular economy, provide a framework for mitigating these future risks. For example, helium reclamation programs and the development of cryogen-free MRI systems are promising solutions, provided they do not depend upon further higher energy requirements.^35^ Raw iodine is another finite resource and extraction is costly. Multidose contrast packaging and delivery systems have been shown to reduce contrast (and packaging) waste,^52^ and contrast recycling is an important complement for unused contrast.^53^

### Green Lean Six Sigma

Green Lean Six Sigma is another economic framework that has been applied to enhance planetary health.^54^ This combines 2 distinct approaches, Lean and Six Sigma, with a planetary lens. Lean strives to eliminate activities that do not add value; Six Sigma seeks to improve quality by reducing variability.^55^ Green Lean Six Sigma promises improved operational efficiency by minimizing unnecessary steps and optimizing resource utilization, patient outcomes, and profitability.^56^^,^^57^ However, Green Lean Six Sigma has not been widely adopted due to complexities in implementation. EcoRad offers a simpler, more cohesive framework that is tailored to radiologists and their practices.

### Value-based care

Value-based healthcare aligns well with EcoRad, as it seeks to economically incentivize improved patient outcomes, efficiency, and sustainability. Value-based care varies among countries. In the United States, the rise of alternative payments models, most notably the Merit-Based Incentive Payment System (MIPS), offers opportunities to incentivize planetary health. US radiologists are at a disadvantage in the current MIPS, due to a paucity of available quality measures.^58^^,^^59^ Nations with value-based reimbursement, such as the UK’s best practice tariffs, present radiologists with an opportunity to propose and validate sustainability-focused quality measures.

## Overcoming economic barriers to sustainable radiology practice

Despite its benefits, EcoRad faces hurdles to adoption due to several biases and competing priorities. Growing sustainable radiology relies on overcoming these challenges.

### Biases

The *status quo* bias, a preference for the current state even when alternatives offer better value, can hinder adoption. To counter this, highlight successes in other organizations and emphasize the potential to enhance reputation and patient satisfaction.^51^ The sunk cost fallacy is the tendency to continue investing in a failing venture due to past investments. This fallacy can prevent departments from abandoning outdated, environmentally damaging equipment and processes.^60^ To counter this, LCA and cost-effectiveness analyses can solidify the joint environmental and financial advantages of sustainable practices.^19^^,^^28^ Finally, prioritizing short-term profit and growth can overshadow the long-term benefits of sustainability. To overcome this, we recommend educating decision-makers on the triple bottom line—patients, planet, and productivity—and integrating these goals into performance metrics.^60^

### The potential benefits and burdens of AI

AI promises economic and environmental benefits through efficiency and reduced waste. Meanwhile the primary liability of AI—substantial consumption of energy and water—continues to grow, driving increased financial and environmental costs.^61^ To overcome this, we propose additionality—matching data centre growth with growth of clean energy.

Another evolving consideration is the role of AI in burnout. Initial enthusiasm centred on AI’s promise to eliminate menial tasks and reduce burnout. However, recent studies suggest that AI applications might aggravate radiologists’ workload.^62^ The economic consequences of burnout are well documented.^63–66^ Furthermore, as AI algorithms become more advanced and institutions’ portfolio of AI applications continue to grow, costs have increased prodigiously.^67^^,^^68^ Consolidating AI models and selecting tasks that can be simplified by algorithms, such as comparing lesion size and summarizing clinical history, can reduce costs and burnout. These economic pitfalls must be recognized for AI to reach its promised potential in furthering sustainability.

### Economic barriers preventing health and environmental equity

Climate change preferentially impacts vulnerable populations, such as children, elders, and those living in poverty.^69^ In turn, economic barriers to build sustainable radiology practices loom larger in these populations.^70^ Applying EcoRad to all is vital. Strategies include improving patient access to radiology services, developing outreach programs to serve “imaging deserts,” and screening for climate resilience.^14^^,^^55^

## Call to action

**Figure 3.**
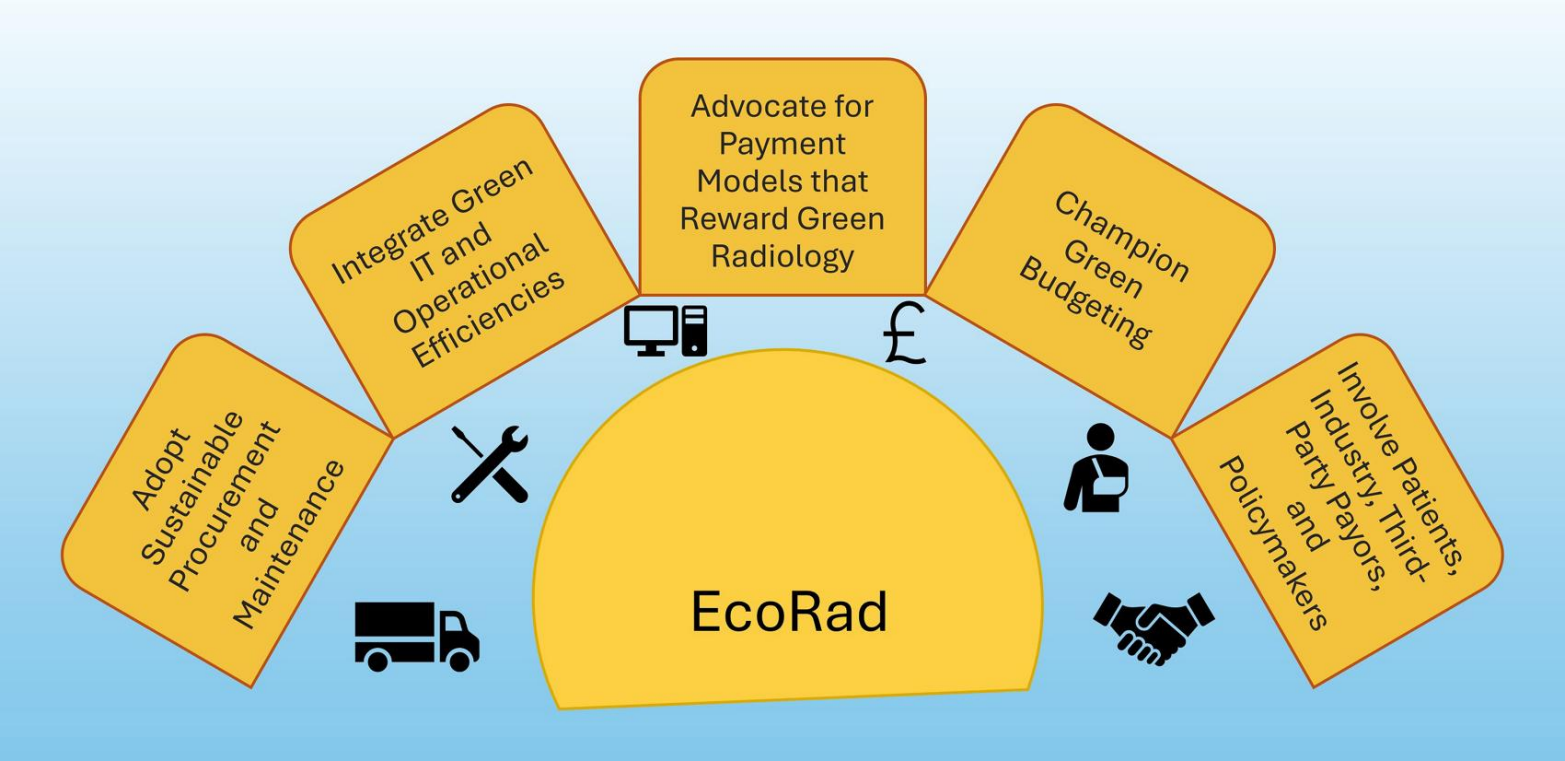
Calls to action: implementing EcoRad.

To implement EcoRad in daily radiology practice, we propose the following 5 calls to action, shown in Figure 3.

### #1—Adopt sustainable procurement and maintenance

Radiology leaders can procure imaging equipment and supplies designed with sustainability in mind. Departments should favour products that are energy-efficient, designed for longevity, made from recyclable materials, and limit waste. Additionally, regular maintenance can extend the life of existing equipment. This reduces the waste and Scope 3 emissions associated with frequent replacements. Furthermore, geopolitical tensions and the spectre of tariffs have incentivized the adoption of circular manufacturing processes. Specifically, as costs to extract and import raw materials increase, value in recirculating components will increase. Radiology could become a leader in the circular economy, which is expected to add $4.5 trillion in economic value per European Parliament and Accenture estimates.

### #2—Integrate green IT and operational efficiencies

Data management is central to modern radiology. Radiology departments should work closely with IT colleagues to implement green practices, such as turning off computers after hours, energy-efficient servers, cloud storage, and power-saving protocols. As data storage requirements grow and increased computing power is necessary, additionality should guide new energy needs—clean energy growth must match data centre growth. In addition to reducing energy waste, improved scheduling represents another opportunity for green IT practices to lower operational costs, while significantly reducing environmental footprint.

### #3—Advocate for payment models that reward green radiology

Due to the outsized impact of radiology on healthcare emissions, radiologists are well positioned to propose innovative payment models. These approaches will reward care that improves quality, optimizes efficiency, maintains patient access, and reduces environmental impact. Radiologists’ involvement in co-developing these models is vital to successfully align these incentives.

### #4—Champion green budgeting

By championing value-based care (based on the alternative payment models above) and incorporating green budgeting, radiology departments can create transparent metrics that track the environmental costs. This will not only improve patient outcomes, but also position radiology departments as leaders in sustainable healthcare.

### #5—Involve patients, industry, third-party payors, and policymakers in sustainability

Integrating stakeholders into our radiology green teams is vital to our shared success. Patients can become informed advocates for green practices and partner with clinicians committed to sustainability. Industry can collaborate with radiology departments to develop eco-friendly technologies.^71^ Woolen et al provide an excellent example of such collaboration,^33^ with results supporting the triple aim of EcoRad—reduced emissions, reduced costs, and improved patient access. Third-party payors can create incentives for sustainable practices through alternative payment models. Policymakers can establish clear and enforceable regulations that drive adoption of green healthcare. These partnerships are vital to foster a greener, healthier, and more economically sound future for radiology.

## Conclusion

In this review, we have redefined “eco-” as a bridge between the natural world and the economic systems that underpin the practice of radiology. We have shown how a planetary lens on radiologic economics can help target cost savings and operational efficiency while advancing the goals of environmental stewardship. Conversely, by applying economic frameworks to environmental challenges, radiology can contribute to more sustainable and resilient healthcare.

Blending ecology and economics under the unified “eco” framework gives rise to EcoRad, a vision to transform the future of radiology. This vision recognizes that every decision made by a department, from procuring new equipment to optimizing daily operations, has both economic and environmental implications. Armed with this knowledge, radiology departments can embrace the practical strategies of EcoRad to enhance operational efficiency, reduce costs, and reduce environmental impact. This will result in healthier patients, healthier communities, and a healthier planet.
